# Quality of life of women from families of martyred individuals in the Kurdistan region of Iraq as a conflict area in the Middle East

**DOI:** 10.1186/s12914-020-00248-6

**Published:** 2020-11-30

**Authors:** Hamdia Mirkhan Ahmed

**Affiliations:** College of Health Sciences, Hawler Medical University, Erbil, Iraq

**Keywords:** Women, Quality, Life, Veteran, War, Conflict, Kurdish

## Abstract

**Background:**

Quality of life (QOL) research develops data and insight into issues that pertain not only to the individual, but that can also apply to the population as a whole. This study aimed to analyze the QOL of Kurdish women from families of martyred individuals in the Kurdistan region of Iraq.

**Methods:**

A cross-sectional study of 380 women from families of martyred individuals was conducted. All women were patients at the Medical Center of Martyr Families in Erbil City from January 2018 to April 2019. Data were collected through interviews and the WHOQOL-BREF scale was used to measure QOL. The women’s QOL scores were divided into four categories (i.e., quartiles): 1st, 2nd, 3rd and 4th quartile. Kruskal-Wallis and Chi-Square tests were used for data Analysis.

**Results:**

The women’s QOL scores fell into the following quartiles: Overall QOL and General Health (*n* = 66.6%) in the 1st quartile, Physical and Psychological Health (*n* = 56.9%) in the 2nd quartile, Social Relationships (*n* = 47.9%) in the 3rd quartile, Environmental health (*n* = 85.6%) in the 2nd and 3rd quartile. The total QOL of more than half (*n* = 52.1%) of the women studied were in 1st and 2nd quartiles.

**Conclusion:**

Women from families of martyred individuals were not satisfied with their QOL, especially in terms of Physical and Psychological Domains. International political and humanitarian actions are needed to reduce the destructive consequences of war and conflict on these suffering women.

## Background

The World Health Organization (WHO) defines Quality of Life (QOL) as “an individual’s perception of their position in life in the context of the culture and value systems in which they live and in relation to their goals, expectations, standards and concerns. It is a broad ranging concept affected in a complex way by the person’s physical health, psychological state, personal beliefs, social relationships and their relationship to salient features of their environment” [1]. Compared to men, women tend to suffer more severely from damage to health infrastructure and economic downturns, as well as from displacement and dislocation during and after conflict [2].

Quality of life research increases knowledge related to the clinical care of individuals, helps to develop an epidemiological perspective on problems, provides data to evaluate the cost-effectiveness of various interventions and prevention strategies, and guides the restructuring of healthcare systems. Thus, QOL data have significant implications for social and public policy [3]. QOL research is needed in Iraq, especially in Kurdistan, given the countries long history of war and conflict, which has led to the massive loss of men from families. Many of these men have died for the principal slogans of the revolution: freedom, social and economic justice, and democracy. According to statistics by the Erbil General Directorate of Martyrs and the Anfal Affairs, there are about 20,000 families of martyrs (i.e., veterans) in Iraq. In the Erbil governorate, there may be one to five martyrs per family. In many of these families, the women assume the responsibility of family support and even custodial family support. In this way, wars and armed struggles in the region have pushed Kurdish women to play multiple and variable roles within a family, changing gender relations and social positions. As a result, women have experienced a novel set of problems and challenges. How women respond to these challenges, oftentimes through activism and other forms of resistance, varies based on the multiple racial, religious, and rural/urban identities of women in different parts of Kurdistan [4].

The Medical Center of Martyr Families was first established in 2000 in Erbil City. This center many of the patients who seek care at this center are middle-aged women or older. While a majority of these women visit the center for physical or mental health treatment, many clearly exhibit the need for psychological and economic support as well, typically due to the loss of the family’s leader and breadwinner (e.g., husbands, fathers, and brothers). As the sole surviving adult, these women are forced to assume all or nearly all family roles. Some women of reproductive age want to remarry or otherwise change their lifestyle, but oftentimes cannot because of traditional and social obstacles of single parenthood. (BQ Saleem, Personal communication, 15 February 2018).

The general purpose of the present study is to conduct a needs assessment of Kurdish women, specifically from families of martyred individuals, who visit this center to better understand the unique health and social challenges that they face in supporting their families. To conduct this assessment, this study used interviews to determine the QOL of Kurdish women affected by conflict. Statistical analysis was used to explore the association of the women’s QOL scores with their sociodemographic characteristics.

## Methods

A cross sectional study was conducted in the Medical Center of Martyr Families in Erbil City from January 2018 to April 2019. Using non-probability sampling, This center is under the direction of the Ministry of Martyrs and Anfal (Genocide), Kurdistan region, Iraq. In the entire Erbil Governorate this is the only medical center available to provide health care specifically for this group and it is only accessible to families living within the city limits. Surgeon, internist, rheumatologist, urologist, dentist, ENT and dermatologist are available in this center. Basic laboratory tests, x-ray and sonography are available; for more advanced intervention, treatment and care, the cases are referred to government hospitals. Three hundred eighty women from families of martyred individuals were recruited from the center for participation in this study. The Epiinfo 7 Computer Program was used to calculate the sample size. The following information was entered into the program: population size (*n* = 50,000), expected proportion of women of quality (*n* = 50%), precision (*n* = 5%), and confidence level (*n* = 95%). The expected proportion of women of quality was set to 50% to account for the unavailability of data, as 50% yields the highest sample size. Based on this input, the estimated sample size was 381. Three-hundred eighty women were considered for convenience. Prior to data collection, permission to conduct this study was secured from the Directorate of Martyrs in Erbil City and the study proposal was approved by the Scientific and Ethical Committee of College of Nursing, Hawler Medical University.

A questionnaire form including demographic characteristics was developed for the purpose of data collection. For measuring QOL, the WHOQOL-BREF scale was used. This scale assesses an individual’s perception of their life in the context of their culture and value systems, their personal goals, and their standards and concerns. The WHOQOL instruments were collaboratively developed in a number of centers worldwide, and have been widely field-tested. The WHOQOL-BREF instrument comprises of 26 items, which measure the following broad domains: physical health, psychological health, social relationships, and environment. The WHOQOL-BREF is a shorter version of the original instrument (WHOQOL-100) that may be more convenient for use in large research studies or clinical trials. Scoring of the scale was done according to WHOQOL-BREF U.S. guidelines [5]. Although the WHOQOL-BREF is a self-administered instrument, the data was collected through direct interviews with the participating women due to high rates of illiteracy. While this instrument is not validated in the Kurdistan region and in the Kurdish language, it is validated in the Arabic language in Jordan, which is also the formal language of Iraq [6].

Informed consent was obtained from the wives, mothers, sisters and daughters who were selected to participate in this study. The purpose of the study was explained and questions were answered by the research staff. All forms were anonymized and were instead assigned a code. Data were entered into and analyzed by the Statistical Package for Social Silences V.23. The Kolmogorov-Smirnov statistical test was used to determine the normality distributions of the quantitative variables. The samples were divided into four categories (i.e., quartiles) according to QOL score: 1st quartile was the lowest 25%; 2nd quartile was the next lowest 25%, up to the median; 3rd quartile was the second highest 25% of numbers, above the median; 4th quartile was the highest 25% of numbers. Frequency, percentage, Kruskal-Wallis, Mann-Whitney, and chi-square tests were all used for data Analysis.

## Results

In total, 380 women from families of martyred individuals engaged in this study. The mean age was 48.6 ± 13.5 years of age and the mean years of education was 3.5 ± 4.7 years. More than half of the study sample (*n* = 51.6%) were aged between 41 and 60 years old and a majority were illiterate (*n* = 53.7%). The majority of women were housewives (*n* = 92.9%) and were married (*n* = 90.5%). Most of the women were either the wife, sister, or daughter of a man who had been martyred (Table 1).

**Table 1:** Sociodemographic characteristics of the study sample

Variables	Values	No	%
Age (years)	≤20	5	1.3
	21–40	111	29.2
	41–60	196	51.6
	> 60	68	17.9
Education level	Illiterate	204	53.7
	Basic	137	36.1
	Secondary	8	2.1
	Institute & above	31	8.2
Occupation	Housewife	353	92.9
	Employee	4.5	4.5
	Other	2.6	2.6
Marital status	Single	36	9.5
	Married/widowed	344	90.5
Relation with martyr	Mother	50	13.2
	Wife	78	20.5
	Sister	113	29.7
	Daughter	139	36.6

The highest percentage (*n* = 66.6, 56.9, and 69.2%) of the women’s overall QOL/general health, physical health, and psychological health were in 1st and 2nd quartiles, respectively. Nearly half (*n* = 47.9%) of the women’s quality of social relationships scored in the 3rd quartile. The majority (*n* = 85.6%) of quality of environment scores were in the 2nd and 3rd quartile. The total QOL for more than half of the women (*n* = 52.1%) were in the 1st and 2nd quartiles (Table 2).

**Table 2:** Frequency (%) of QOL domains of the study sample based on Quartiles of WHOQOL-BREF score

Domains	Q1 (0–25%)	Q2 (2 5-50%)	Q3 (50–75%)	Q4 (75–100%)
Overall QOL and general health	137 (36.1)	116 (30.5)	118 (31.1)	9 (2.4)
Physical health	123 (32.4)	93 (24.5)	100 (26.3)	64 (16.8)
Psychological	131 (34.5)	132 (34.7)	81 (21.3)	36 (9.5)
Social relationship	6 (1.6)	21 (5.5)	182 (47.9)	171 (45)
Environment	27 (7.1)	142 (37.4)	183 (48.2)	28 (7.4)
Total QOL	35 (9.2)	163 (42.9)	142 (37.4)	40 (10.5)

*Q* Quartile, *QOL* Quality of life

A highly statistically significant association was found between the women’s level of QOL and their age, educational level, occupation, marital status, and their relation to a martyr. Among age groups, more than half of women 21–40 years old were in the in the 3rd quartile in terms of quality of life, and more than half of women over the age of 60 were in the 2nd quartile in terms of quality of life. Women who were illiterate or had a basic education level had a lower quality of life score. Nearly half of the women who scored under the median in terms of quality of life were housewives, married, or widowed. More than half of the women in the 2nd quartile for quality of life were mothers or wives of martyrs. (Table 3).

**Table 3:** Association between total QOL of the study sample with their socio-demographic characteristics

Variables	Values	Q1 (0–25%)	Q2 (25–50)	Q3 (50–75%)	Q4 (75–100%)	***P***-value
Age (years)	≤20	0 (0)	0 (0)	1 (20)	4 (80)	< 0.001
	21–40	5 (4.5)	22 (19.8)	61 (55)	23 (20.7)	
	41–60	15 (7.7)	103 (52.6)	66 (33.7)	12 (6.1)	
	> 60	15 (22.1)	38 (55.9)	14 (20.6)	1 (1.5)	
Education level	Illiterate	23 (11.3)	116 (56.9)	59 (28.9)	6 (2.9)	< 0.001
	Basic	12 (8.8)	44 (32.1)	67 (48.9)	14 (10.2)	
	Secondary	0 (0)	1 (12.5)	5 (62.5)	2 (25)	
	University	0 (0)	2 (6.5)	11 (35.5)	18 (58.1)	
Occupation	Housewife	35 (9.9)	161 (45.6)	132 (37.4)	25 (7.1)	< 0.001
	Employee	0 (0)	1 (5.9)	8 (47.1)	8 (47.1)	
	Other	0 (0)	1 (10)	2 (20)	7 (70)	
Marital status	Single	0 (0)	5 (13.9)	16 (44.4)	15 (41.7)	< 0.001
	Married/widowed	35 (10.2)	158 (45.9)	126 (36.6)	25 (7.3)	
Relation with martyr	Mother	11 (22)	27 (54)	11 (22)	1 (20)	< 0.001
	Wife	9 (11.5)	40 (51.3)	28 (35.9)	1 (1.3)	
	Sister	8 (7.1)	51 (45.1)	43 (38.1)	11 (9.7)	
	Daughter	7 (5)	45 (32.4)	60 (43.2)	27 (19.4)	

*Q* Quarter

When asked, “Do these questions show your quality of life?”, a majority of women (*n* = 99.2%) responded “Yes.”

**Fig. 1:**
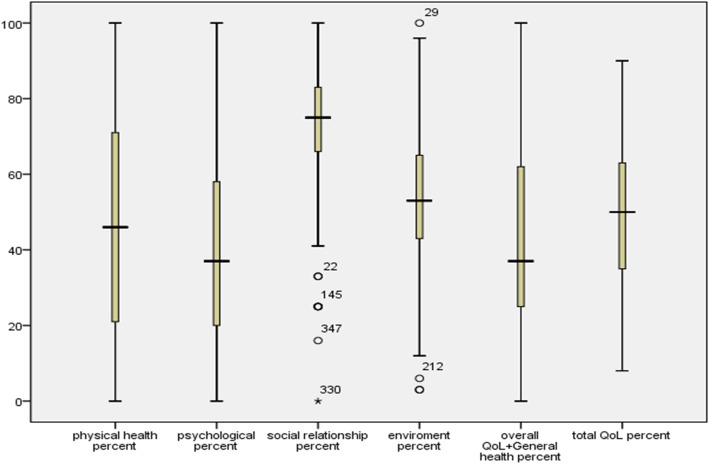
Box plot indicating median, quartiles and extreme values for domains of QOL

The median for the social relationship domain of QOL was found to be higher than the median of other domains. The psychological domain of QOL and overall QOL/general health had the lowest medians (Fig. 1).

## Discussion

The present study examined the QOL of 380 Kurdish female relatives of martyred individuals. Each of the women included in this study lost a male family members (e.g., husbands, sons, brothers, or fathers) in the last 50 last years. These men, called Peshmerga, died defending Kurdistan from threats by Iraqi regimes and attacks of neighboring countries. Some were the victims of the 1970 genocide, while others were war prisoners who were hung or tortured to death. Still others left as part of the Kurdish exodus in 1991 or were killed during the fight against Da’ish, the Islamic State’s (i.e, ISIS) militant group [7, 8]. According to their WHOQOL-BREF score, the results of this study show that the QOL of surviving women in this region is not desirable, even poor, depending on age, educational level, occupation, marital status, and relationship to the martyr. Women of older age, low educational status, unemployed and married/widowed women had a lower QOL, as well as those women who were the mother or wife of the martyr. The quality of physical health and psychological health of the study sample were low, which may be due to lack of support by governmental health organizations and the lack of provision of healthcare to this group of women, as well as limited financial support. In addition, sociodemographic factors – especially low economic status and lack of education—may further worsen QOL for these women, as challenges such as illiteracy complicate everyday life.

Other studies have shown that QOL deficits in both veterans and refugees have been consistently linked to war-related post-traumatic stress symptoms [9, 10]. One study done in Western Europe on Balkan residents and refugees showed that employment and finances were among the strongest factors of dissatisfaction by participants and that social QOL (SQOL) was strongly affected by post-traumatic stress symptoms. Lower SCOL were directly dependent on traumatic war events and post-war environment [11].

Further, the results of the present study show that quality of the social relationship domain of the interviewed women was higher than other domains. This may be due to strong social relationships among Kurdish people, specifically in the Mediterranean East countries. Culturally, the relationship between family members and relatives is strong and there tends to be a strong sense of religious heritage. What’s more, close family relationships among Kurdish people are an important part of traditional value systems. Typical family structures in this region include extended family members and it is very uncommon for Kurds to live alone. Families tend to be large, as several generations of affiliated family members tend to live together. Family networks remain tight even in cases where family members live far from one another [12]. In nuclear and extended Kurdish families, familial loyalty, kinship, strong marriage relationships, cooperation in work, and strong support systems are the norm. Large kin groups are of higher importance than ethnicity, social class, and sectarian lines. Further, family members are mutually protective of one another. An individual’s status within a family group is determined by the family’s position and the individual’s position within that group [13]. As the vast majority of Kurdish people are Muslim, family is of utmost importance and the family unit is regarded as the cornerstone of a healthy and balanced society [14].

Given the importance of stable family structures, the Kurdistan Region Government (KRG) has tried to support those families who have lost members to conflict, especially those who lost their breadwinners. Support includes a monthly salary and support for healthcare, education, and housing [15]. Despite this support, it seems that these families continue to struggle to meet basic needs. As the results of this study show, only 10.5% of the study sample scored in the 4th quartile of total QOL according WHOQOL-BREF. It is worth mentioning that the poorest families of individuals who had been martyred attend the Medical Center of Martyr Families to seek healthcare services. However, results of this qualitative study indicate that women were not satisfied with the health services offered at this center. Specifically, they expected that more assistance would be provided from the government, and that services would respect them and provide emotional, social, economic, and physical support [16].

There are a number of limitations to this study. While prior studies have assessed quality of life among specific groups of women with particular diseases, such as those with cancer, age-related illnesses, and conditions such as menopause, few-to-no studies have addressed how war affects these populations. As thus, there was limited literature to support this study. Another limitation of the present study was interviewing women who live inside Erbil City, the capital of the Erbil governorate. Many families of martyred individuals live in small towns and villages, which were not included in this study. These women may have worse health than those included in the study because they have less access to adequate healthcare. Another major limitation of the present study is the absence of a control group, which should be included in future studies of this nature.

## Conclusions

Women of families of martyred individuals were not satisfied with their quality of life, especially in terms of quality of physical and psychological health. A low sociodemographic status can make quality of life even worse, which intensifies the adverse outcomes of military conflicts. Governmental support in providing special healthcare and financial support to war veterans is necessary. International political action is further needed to eradicate the effects of war and conflicts so to reduce the suffering of women in conflicted-affected regions.

## Data Availability

The datasets used and/or analyzed for this study are available by the corresponding author upon reasonable request.

## Abbreviations

KRI: Kurdistan Region Iraq
Q: quartile
QOL: Quality of Life
SOQL: Social Quality of Life
WHO: World Health Organization
WHOQOL-BREF: A shorter version of the original instrument of WHOQOL-BREF
